# Emergent Mechanisms in Biocatalysis

**DOI:** 10.1021/acscentsci.5c00245

**Published:** 2025-06-05

**Authors:** Felix C. Raps, Todd K. Hyster

**Affiliations:** † Department of Chemistry, 6740 Princeton University, Princeton, New Jersey 08544 United States

## Abstract

Enzymes are invaluable tools for solving challenges in synthetic organic chemistry. Beyond replicating native reactivity patterns, modern directed evolution strategies have enabled chemists to efficiently survey chemical space to identify enzyme families capable of catalyzing non-natural reactions. While methods often focus on chemo-, enantio-, and regiocontrol, there are a growing number of examples that describe reactivity patterns and reaction mechanisms that were previously unknown in the synthetic literature. In this Perspective, we will explore examples of such emergent mechanistic pathways of enzymes in the context of synthetic precedents, emphasizing the remarkable versatility of diverse enzyme active sites in controlling unprecedented transformations.

## Graphical abstract

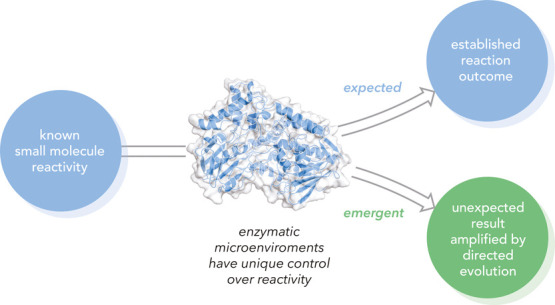

Enzymes have continuously served as a reservoir of inspiration for researchers eager to understand and emulate Nature’s incredible catalytic power.^1^ To this end, chemists have developed small molecule catalysts that simplify biological cofactors or mimic catalytic folds in enzymes.^2,3^ These molecules not only recapitulate native enzyme functions but expand on their capabilities by accepting a more diverse set of substrates and even catalyzing transformations that are unknown in nature.^4,5^ These studies, in part, helped to popularize the notion that small molecule catalysts were more versatile than enzymes. However, studies over the past two decades, spurred by developments in next-generation sequencing,^6^ microfluidics,^7^ high-throughput analytical techniques, and DNA synthesis,^8^ have revealed that some enzyme families can catalyze reactions beyond their native function.^9^ These enzymes can be optimized to catalyze non-natural reactions with levels of selectivity that surpass what is observed with their small congeners.

When developing novel functions for enzymes, most researchers recapitulate reactivity patterns already known in the small molecule catalysis literature.^10–12^ To that end, *de novo-*designed enzymes or substrate promiscuous native enzymes are often selected because of their tailored active sites.^13^ With its myriad noncovalent interactions, the active site can alter the reactivity of catalytic intermediates in distinct ways.^14^ While directing the enantio-, diastereo-, and regioselectivity are favored outcomes for synthetic chemists, the protein microenvironment can also control the energetic profile of reactive intermediates to unlock new reaction mechanisms that are not available to biomimetic small molecule catalysts. Such rare examples of mechanistic emergence highlight the unique reactivity available when transformations occur within well-defined microenvironments. These new functions can accelerate the synthesis of societally valuable molecules and expand our understanding of the mechanisms available to particular molecules and reactive intermediates.^15^

This outlook will cover examples of mechanistic emergence in non-native biocatalytic transformations. We will focus on two types of emergence: (i) cases where enzymes catalyze reactions using a mechanism that is unique to the protein with no corollary in the small molecule or enzyme catalysis literature (*type 1*), and (ii) examples where enzymes catalyze a new transformation that is unknown in the synthetic literature (*type 2*). In doing so, we hope to highlight some of the unique capabilities of enzyme environments for organic synthesis and provide an outlook on the growing diversity in biocatalysis.

## Metalloenzymes

Metalloenzymes have been favored protein scaffolds for non-natural reactivity because metallocofactors have served as inspiration when developing organometallic catalysts. Iron porphyrin and salen complexes were created,^16,17^ in part, to better understand and expand on the ability of cytochrome P450s to catalyze oxygen insertion into C–H bonds and olefins.^18^ Beyond oxene transfer, researchers found that these small molecule catalysts can catalyze isoelectronic carbene and nitrene transfer reactions.^19^ For instance, Kodadek and Woo demonstrated that iron tetratolyl porphyrin (FeTTP) could catalyze carbene transfer to styrenes using ethyl diazoacetate as a carbene precursor.^20^

Inspired by this catalytic versatility of iron porphyrins and early metabolic studies by Mansuy and de Montellano on carbene and nitrenes in the context of cytochrome P450,^21–24^ Arnold and co-workers explored the ability of P450s to catalyze carbene and nitrene transfer reactions (Figure 1).^25^ In an early study, they found that variants of the P450 from *Bacillus megaterium* (P450 BM3) could cyclopropanate styrenes using ethyl diazoacetate, with mutations to the protein altering the enantio- and diastereoselectivity.^25^ Concurrent studies by Fasan et al. demonstrated that heme-dependent myoglobin, an oxygen-binding protein, could also catalyze cyclopropanations.^26^ Subsequent studies have shown the catalytic versatility of heme-dependent proteins for solving selectivity challenges for various carbene and nitrene transfer reactions.^27^

**Figure 1:**
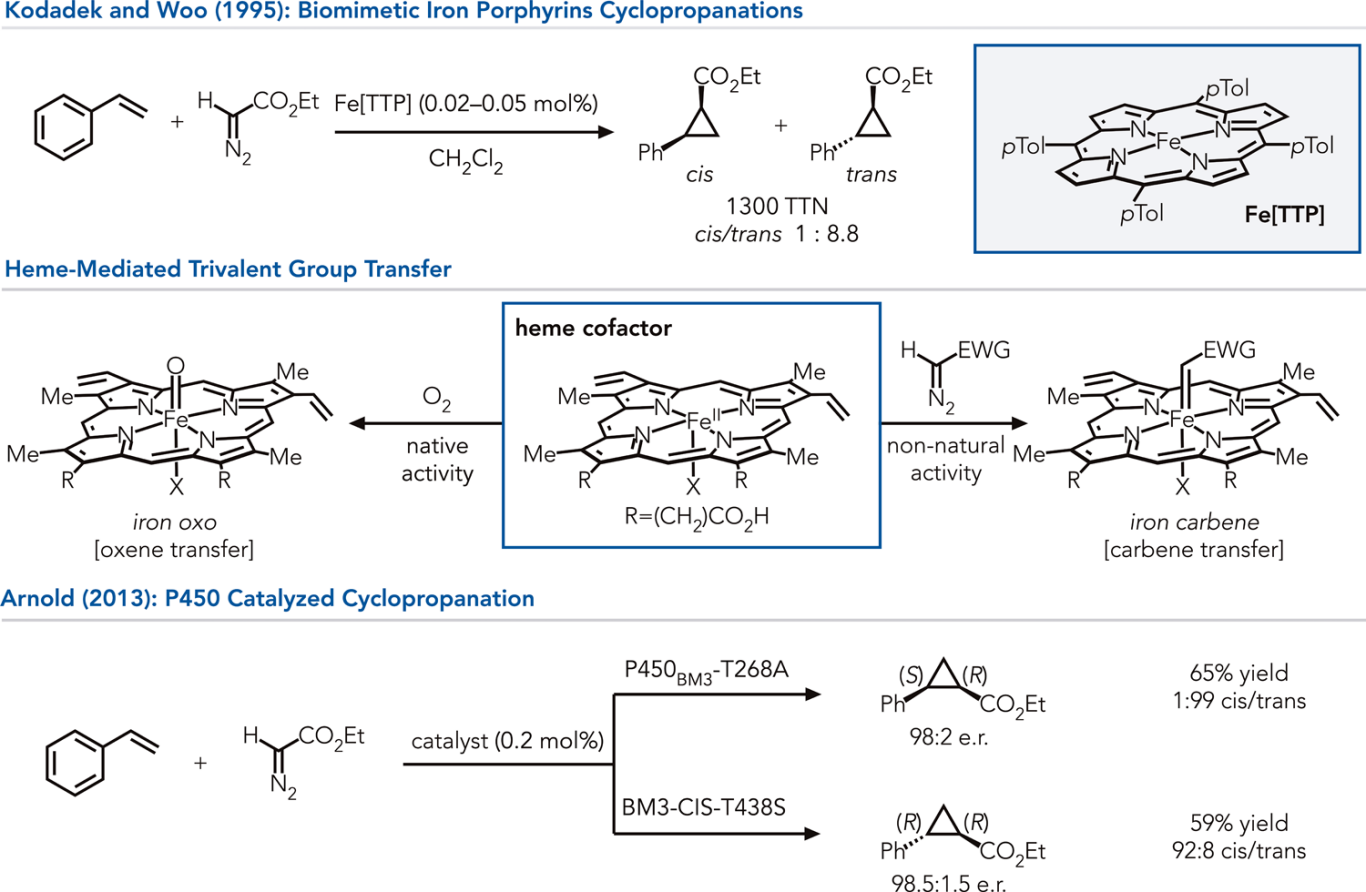
Discovery of alkene cyclopropanation catalyzed by an iron porphyrin led to the development of P450-catalyzed cyclopropanation.

Iron porphyrins have a limited ability to catalyze carbene insertion into C–H bonds.^28,29^ While rhodium and iridium catalysts can insert a wide range of carbenes into various C–H bonds,^30,31^ iron porphyrins are only known to catalyze insertions with donor/acceptor and acceptor/acceptor carbenes (Figure 2).^28^ This limitation is due to the enhanced reactivity of acceptor carbenes, which results in C–H insertion being slow compared to undesired side reactions such as dimerizations.^32^ Arnold and co-workers engineered a heme-dependent protein to catalyze C–H insertion reactions to address this limitation.^33^ After screening a panel of 78 P450s, globins, and cytochrome c proteins, they found P411 *P*4̅(A82L), a catalyst originally engineered for nitrene transfer to thioesters, afforded 13 catalyst turnovers. In addition to the active site and surface mutations, the conserved cysteine, which serves as the axial ligand for heme is mutated to serine, resulting in a less reducing metal center and shift in the Soret band from 450 to 411 nm. Notably, the wild-type P450 BM3 was unreactive, highlighting the importance of these mutations for this emergent function (Figure 2).

**Figure 2:**
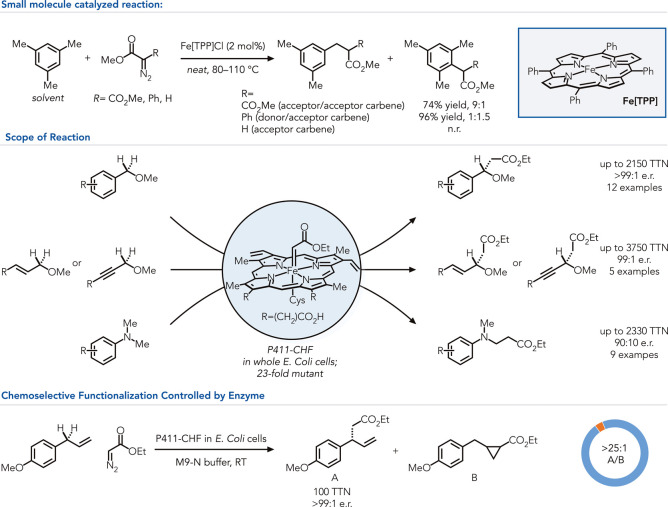
Acceptor carbene insertion into C–H bonds is enabled by protein engineering. Blue = C–H insertion product; orange = cyclopropanation product. n.r. = no reaction.

This burgeoning reactivity was improved using iterative rounds of site saturation mutagenesis effectively amplifying this mechanistic pathway (***type 1***).^34^ The primary protein engineering strategy targeted residues within the active site for site saturation mutagenesis. However, they also found that truncating the FAD binding motif in the reductase domain of the protein increased the activity for C–H functionalization. After 15 rounds of engineering, the final variant (P411-CHF) catalyzed the carbene insertion into the benzylic C–H bond of *p*-OMe benzyl methyl ether with 2’020 total catalyst turnovers with 96.7:3.3 e.r. This enzyme is effective at inserting carbenes into a range of benzylic, allylic, and α-amino C–H bonds. Interestingly, the final variant does not catalyze olefin cyclopropanation, typically the favored reaction with other heme-dependent proteins and biomimetic catalysts. A congener to the model substrate bearing an alkene moiety indicated low formation of cyclopropanated product, overwhelmingly producing the C–H inserted product (>25:1). This work demonstrates the ability of the protein active site to precisely control the energetic landscape of carbene transfer reactions to facilitate reactions that are not observed with simple iron porphyrins.

In a different study, Arnold and co-workers focused on using heme-dependent proteins to prepare cyclopropenes from phenylacetylenes and diazoacetates (Figure 3).^35^ This family of reactivity had been previously reported with [Ir], [Rh], and [Au] complexes, however, these reports are limited to aliphatic alkynes because the arylcyclopropynes readily isomerize to the corresponding furan.^36–39^ In their initial tests, they observed furan byproduct, however, select enzymes produced substituted bicyclobutane, the product of cyclopropanation of the cyclopropene. This is remarkable reactivity because of the instability of the arylcyclopropene, suggesting that the protein is either stabilizing the product within its active site or catalyzing the second cyclopropanation reactions faster than the unimolecular rearrangement of the cyclopropene. The enzyme-controlled process represents unprecedented reactivity and can be described as ***type 2***. Three rounds of iterative site saturation mutagenesis afforded a variant that produces the bicyclobutane product with 1’880 catalyst turnovers. This study highlights the opportunity for optimized proteins to use highly reactive intermediates incompatible with small molecule catalytic systems. One could envision developing enzyme cascades that exploit highly reactive intermediates incompatible with the reaction rates observed with traditional small molecule catalysts.

**Figure 3:**
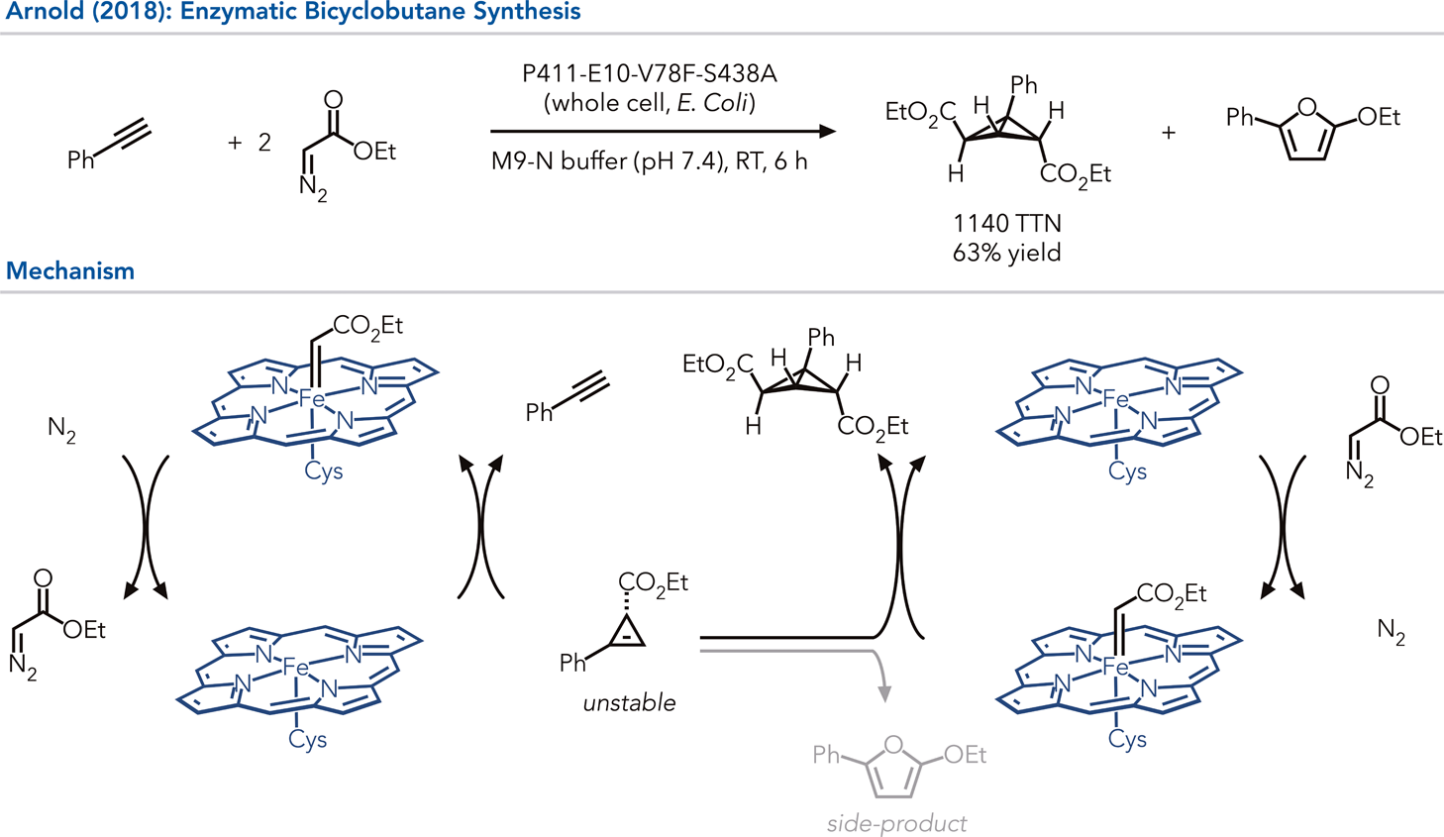
Bicyclobutane synthesis by unprecedented dicyclopropanation of alkynes. The heme cofactor is presented in a simplified version.

In comparison to their carbon congener, iron nitrene species were described in P450 enzymes by Breslow and co-workers in 1982, and later on with Fe­(TPP) species by Mansuy and co-workers in 1984 (Figure 4).^40,41^ These studies served as the starting point for nitrene reactions with iron catalysts for a plethora of transformations.^42^ Nevertheless, both small molecules and natural P450 enzymes mostly rely on the activation hydroxylamine species by prior installation of a leaving group to generate nitrene intermediates. More recently, Arnold and co-workers disclosed a study on nitrene formations for C–H functionalization’s and alkene aminations.^43^ The method overcomes the limitation of substrate activation by generating a precise microenvironment for the hydroxy group to be activated as a leaving group, a feat inaccessible to date in small molecule systems (***type 1***).

**Figure 4:**
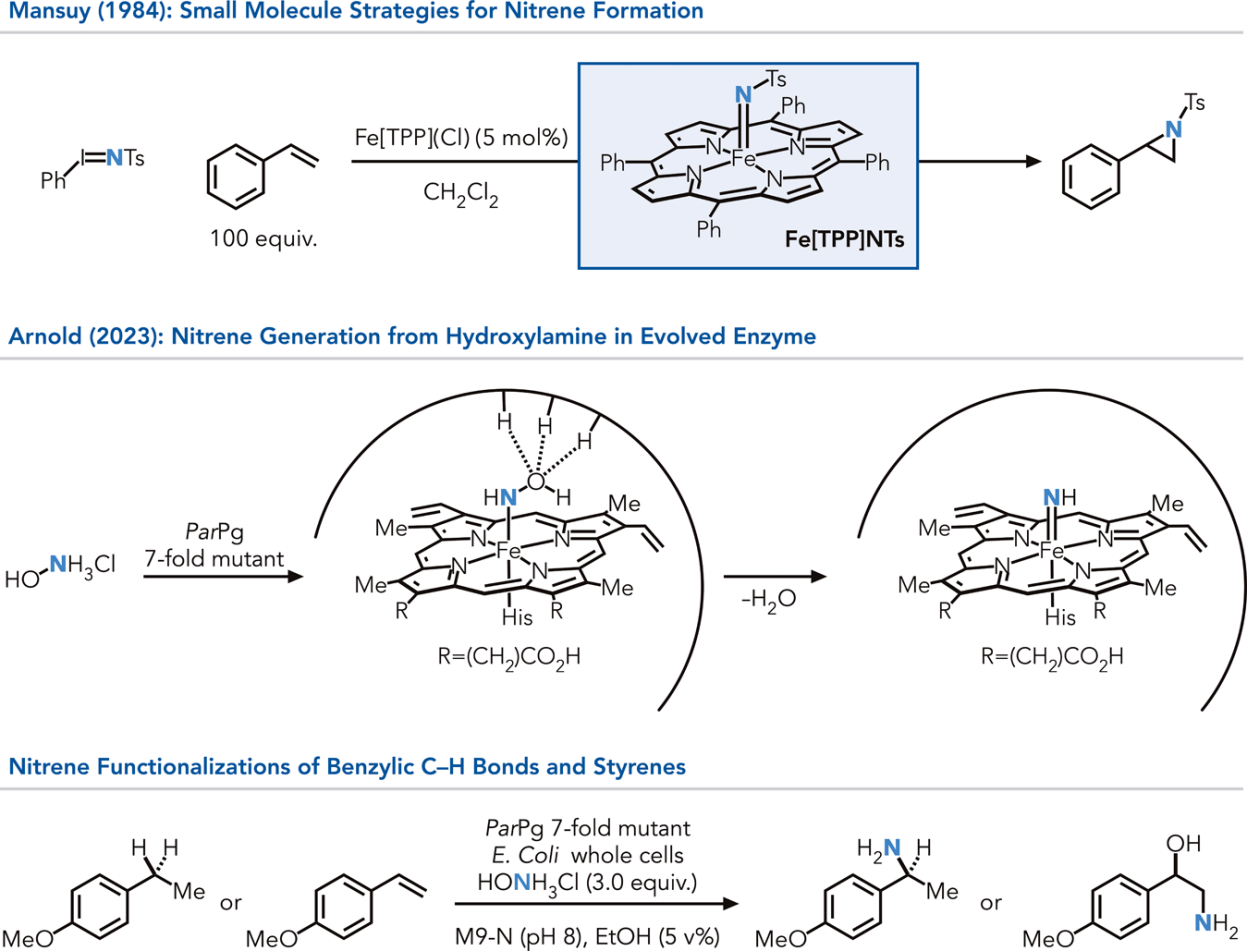
Iron nitrene-based transformations extended to hydroxylamine precursors enabled by a distinct enzyme environment.

Nonheme iron enzymes are increasingly crucial for selective C–H oxidation and halogenation reactions. As a function of having multiple open coordination sites, they are also attractive candidates for catalyzing non-natural reactions.^44^ Recently, Huang and co-workers demonstrated that the (4-hydroxyphenyl)­pyruvate dioxygenase from *Streptomyces avermitilis* (*Sav* HppD) can catalyze an asymmetric group transfer reaction that was previously unknown to small molecule iron catalysts (Figure 5).^45^ In an early study, Cook and co-workers demonstrated that iron triflates can catalyze the isomerization of *N*-fluoroamides to afford benzyl fluorides.^46^ Mechanistically, this reaction begins with cleavage of the N–F bond to form an amidyl radical and Fe–F bond. 1,5-Hydrogen atom transfer produces a benzylic radical, which engages in a barrierless fluorine atom transfer from the Fe–F complex.^46^ Huang found that *Sav* HppD can initiate the same reaction but facilitate alternative group transfers to the benzylic radical going beyond small molecule precedent (***type 2***). Their initial study found that the azide can be transferred to the radical, with the wild-type enzyme affording 9:1 chemoselectivity for azide over fluorine atom transfer. An evolved enzyme variant provided the model azide product with good catalyst proficiency (10’060 TTN) and selectivity (96.5:3.5 e.r.). Computation studies showed that the barriers to fluorine and azide transfer are low (<5 kcal/mol) but that steric constraints imposed by the active site favor azide transfer. This observation highlights the ability of enzymes to distinguish between two energetically similar termination mechanisms with early transition states, a class of reaction that is difficult to control using small molecule catalysts.

**Figure 5:**
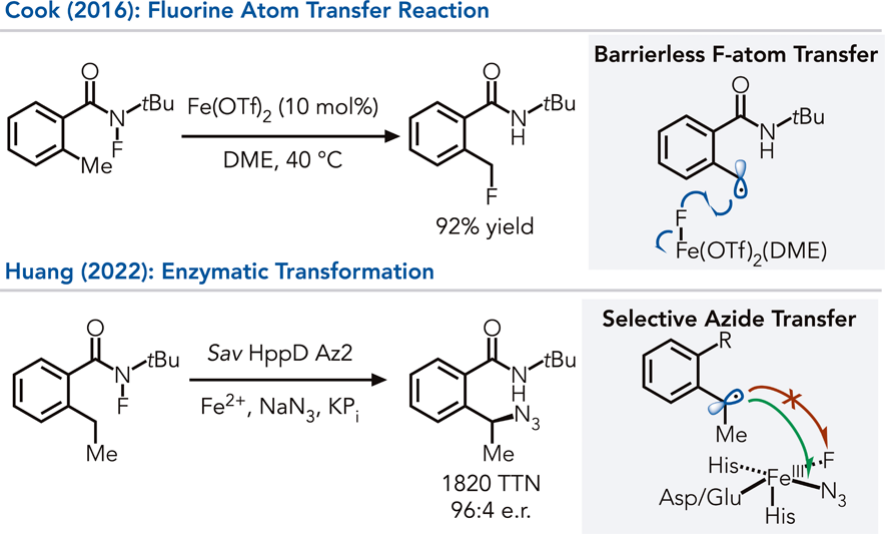
Selective group transfer using nonheme iron enzymes.

## Photoenzymes

The advent of modern methods of radical formation has led to the development of remarkable reactions involving open-shell, free-radical intermediates.^47,48^ An ongoing challenge is to develop catalysts capable of controlling these reactive intermediates. While merging photoredox catalysts with organocatalysts and transition metal catalysts has provided some elegant solutions,^49^ there remain outstanding synthetic challenges, such as reactions that form distal stereocenters or involve substrates lacking traditional catalyst binding handles.

There remain outstanding synthetic challenges, such as reactions that form distal stereocenters or involve substrates lacking traditional catalyst binding handles.

Hyster and co-workers hypothesized that protein active sites could control the stereochemical outcome of radical reactions involving substrates lacking traditional binding handles because of the multitude of noncovalent interactions involved in enzyme catalysts. They began by exploring the asymmetric hydrodehalogenation of alkyl halides. Fukuzumi had previously demonstrated that benzyl bromide can be hydrodehalogenated when *N*-benzyl dihydronicotinamide is excited with near-UV light.^50^ Based on this understanding, they explored nicotinamide-dependent ketoreductases (KREDs) as catalysts for asymmetric hydrodehalogenation of α-bromolactones.^51^ They found that using blue LEDs (460 nm), KREDs with large active sites could catalyze the dehalogenation in high yield with excellent enantioselectivity. While the initial hypothesis was that radical initiation occurred via direct excitation of the NADPH cofactor, this mechanism was ruled out because the reaction did not proceed without the protein. Instead, they found that the reaction is initiated via excitation of a charge transfer complex involving the substrate and NADPH. As these two substrates do not associate with one another in solution, the protein scaffold is required to template this interaction constituting an emergent feature (***type 1***). This serendipitous discovery ensures that radical formation does not occur outside the active site.

Building on this study, Hyster explored flavin-dependent ‘ene’-reductases (EREDs) as photoreductants based on the similarity in structure of flavin hydroquinone and dihydroacridine photoreductants (***type 1***).^52^ They found that the ERED from *Gluconabacter oxidans* (*Glu*ER) could catalyze a 5-*exo*-*trig* cyclizations of α-chloroamides with excellent yield and enantioselectivity when irradiated with cyan LEDs (497 nm). As with KREDs, this reaction is initiated via the excitation of an enzyme-templated charge transfer complex involving flavin hydroquinone and the substrate. Interestingly, these reactions produce very little hydrodehalogenated product, the main product formed when using traditional reagents for radical cyclizations, suggesting that the CT complex only forms in a binding mode that primes the substrate for cyclization (Figure 6).^53^

**Figure 6:**
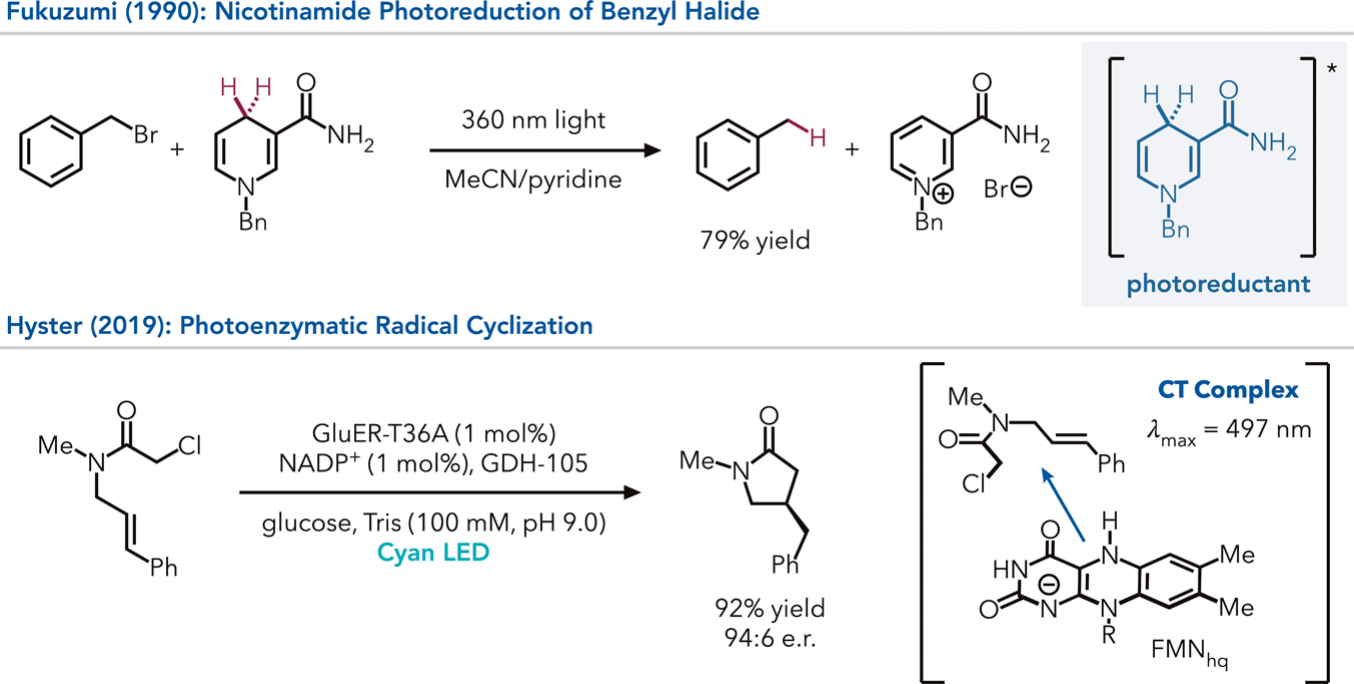
Selective transformation of α-chloroamides into the corresponding lactam control. In contrast to small molecule-catalyzed transformations, hydrodehalogenation can be avoided through enzymatic control.

Next, this ERED reactivity was expanded to intermolecular reactions (Figure 7).^54^ This family of reactivity poses a significant synthetic challenge because of the short lifetime of radical intermediates within photoenzymes.^55–57^ If both coupling partners are not bound within the active site prior to radical initiation, the alkyl halide will be consumed via hydrodehalogenation. However, they found that *Glu*ER can catalyze the coupling of α-chloroamides with α-methylstyrene in good yield and excellent enantioselectivity, forming negligible amounts of the hydrodehalogenated side product. The lack of hydrodehalogenated products can be explained through an emergent phenomenon observed in the charge transfer complexes. When the α-chloroamide is added to the reduced enzyme, a weakly absorbing charge transfer complex is observed. However, a much more strongly absorbing complex is formed upon adding the alkene, offering better quantum efficiencies. Hence, it was determined that the enzyme is templating a higher-order complex between the α-chloroamide, alkene, and FMN_hq_. These higher-order complexes are elusive to small-molecule catalysis because of the entropic penalty and the lack of strong noncovalent interactions between the three molecules (***type 1***).

**Figure 7:**
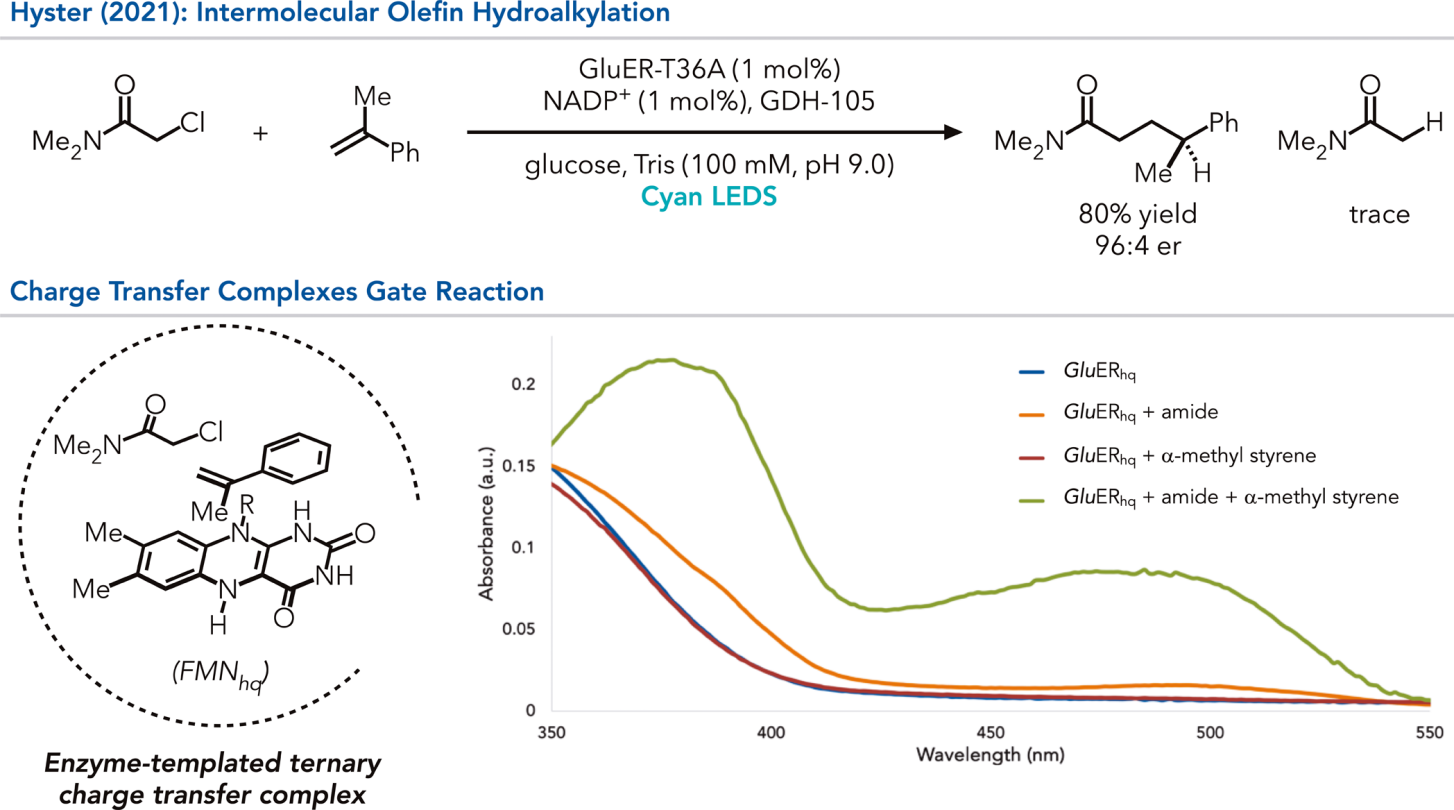
Intermolecular radical C–C bond construction in ene reductases by radical formation gating through charge transfer complexes.

In subsequent studies, they found that the protein structure can strongly influence the degree of coupling and wave function overlap between the molecules.^58^ The microenvironment can be tuned using protein engineering to enable electron transfer reactions to operate in the dark, an unknown phenomenon in the existing CT complex literature. The formation of charge transfer complexes enabled by the protein active site confinement and organization is an emergent feature that extended the capabilities of photoenzymatic catalysis and, in combination with studies by Zhao,^59^ as a starting point for many developments within the field.^60^

Building on the intermolecular coupling reactions enabled by enzyme-templated charge transfer complexes, Hyster and co-workers investigated the coupling of alkyl halides with nitronates to produce β-nitroamides (Figure 8).^61^ Surprisingly, the ‘ene’-reductase from *Caulobacter segnis* (*Cs*ER) could reductively couple these two reagents with loss of the nitro group in good yield with excellent control of the β-stereocenter. This constitutes an unexpected Csp^3^-Csp^3^ reductive cross-coupling. Reductive cross-couplings are an increasingly important family of reactivity because they synthesize complex molecules without requiring organometallic reagents.^62^ Pioneering work by Weix and others demonstrated that nickel can catalyze the Csp^2^-Csp^3^ reactions with excellent cross-selectivity.^63^ While there are examples of Csp^3^-Csp^3^ couplings using different alkyl halides, they are often plagued with significant formation of homocoupled products.^64^ Consequently, observing a reductive coupling reaction using enzymes provides a unique and unexpected solution to this challenge.

**Figure 8:**
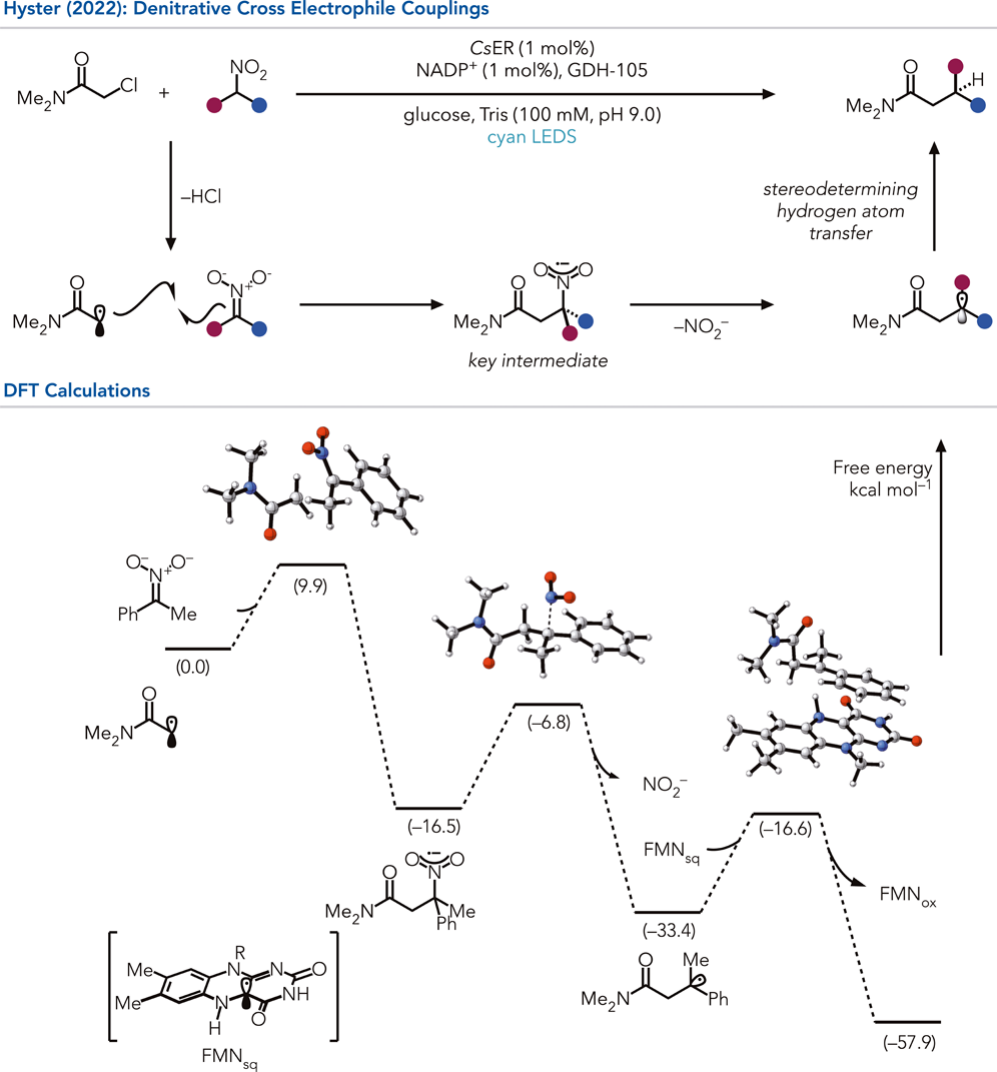
Unlocking of denitrative C–C cross-electrophile couplings via charge transfer complex gating of the intermediate in the enzyme active site.

The central question in this work was the mechanism by which the nitro group is removed in the reaction. The authors hypothesized that the nitroradical anion was a key intermediate. In transition metal-catalyzed reactions, these intermediates are proposed to be potent single electron reductants.^65^ However, early studies by Kornblum and others found that nitroradical anions can undergo mesolytic cleavage to break the C–N bond.^66^ This was further investigated by DFT calculations, which showed that the mesolytic cleavage event was thermodynamically downhill (16.9 kcal mol^–1^) (Figure 8). Since this reactivity had not been observed outside an enzyme active site, verified by control experiments, the reactivity had to depend on the correct enzyme active site environment (***type 2***). As evidence that the protein active site facilitates the scission step, the engineered homologue *Gk*OYE favors electron transfer over mesolytic cleavage.^67^

This work highlights the opportunity for proteins to exploit mechanistic steps largely overlooked in small molecule catalysis because they are understood to be slow compared to other seemingly favored pathways.

Radical C–N bond-forming reactions are essential reactions in modern organic synthesis. To this end, there are multiple reports of olefin hydroamination using highly reactive *N*-centered radicals or olefin radical cations.^68–73^ Alternatively, amines and amides can be coupled to carbon-centered radicals under the aegis of transition metal catalysis or via radical/polar crossover reactions.^69^ Hyster and co-workers described a biocatalytic method to forge C–N bonds that use an alternative mechanism (Figure 9).^74^ While exploring asymmetric hydroaminations, they found that a flavin-dependent Baeyer–Villiger monooxygenase from *Acinetobacter calcoaceticus* (*Ac*CHMO-M10)^75^ could catalyze a 5-*exo*-*trig* hydroamination using a styrenyl alkene and unactivated aniline. While the initial enzyme offered low yields and poor enantioselectivity, five rounds of iterative site saturation mutagenesis increased the enzyme performance to form the product in 95% yield and 95:5 e.r. The 5-*exo*-*trig* product was unexpected because existing radical hydroaminations using photosensitizers produce the 6-*endo*-*trig* product.^76^ The precedented 6-*endo*-*trig* selectivity can be rationalized by the higher stability of the intermediate benzylic radical over the transient methylene radical formation.^77^ To understand the observation of putatively unfavorable 5-*exo*-*trig* product formation, the authors conducted mechanistic studies. They found that the reaction initiates via the reduction of the substrate rather than the oxidative mechanism responsible for hydroamination in the radical literature.^76^ Although flavin hydroquinone (FAD_hq_) is not sufficiently reducing (E^red,^*= – 2.26 V versus SCE) outside of the product to minimize the styrenyl moiety (E^red^= – 2.6 V versus SCE), the protein environment can stabilize negative charge to attenuate that potential.^78^ It was hypothesized that the benzylic radical is oxidized (E^ox^= 0.16 V versus SCE) to the corresponding cation for C–N bond formation. However, this oxidation would be impossible for the flavin semiquinone (FAD_sq_, E^ox^= – 0.25 V versus SCE). Griller et al. described the oxidation potentials of various radical species that overlap with electron density through bond conjugation, which can drastically attenuate the oxidation potential.^79^ It was thus hypothesized that the preorganization of the enzyme could attenuate the oxidation potential through the through-space interactions of the radical and the nitrogen lone pair. DFT investigations showed a drastic lowering of the oxidation potential in a prealigned form. The putative 2-center-3-electron interaction could then be oxidized by the flavin semiquinone and, in turn, form the observed α-tertiary amine product. This conformer was also found by molecular dynamics (MD) and QM/MM simulations, which revealed an increasing population of the expected prealigned conformers throughout the evolution campaign. The hydroamination reactivity under photoredox conditions is solely observed within the enzyme environment and constitutes a distinct reactivity with no example in the small molecule literature (***type 2***).

**Figure 9:**
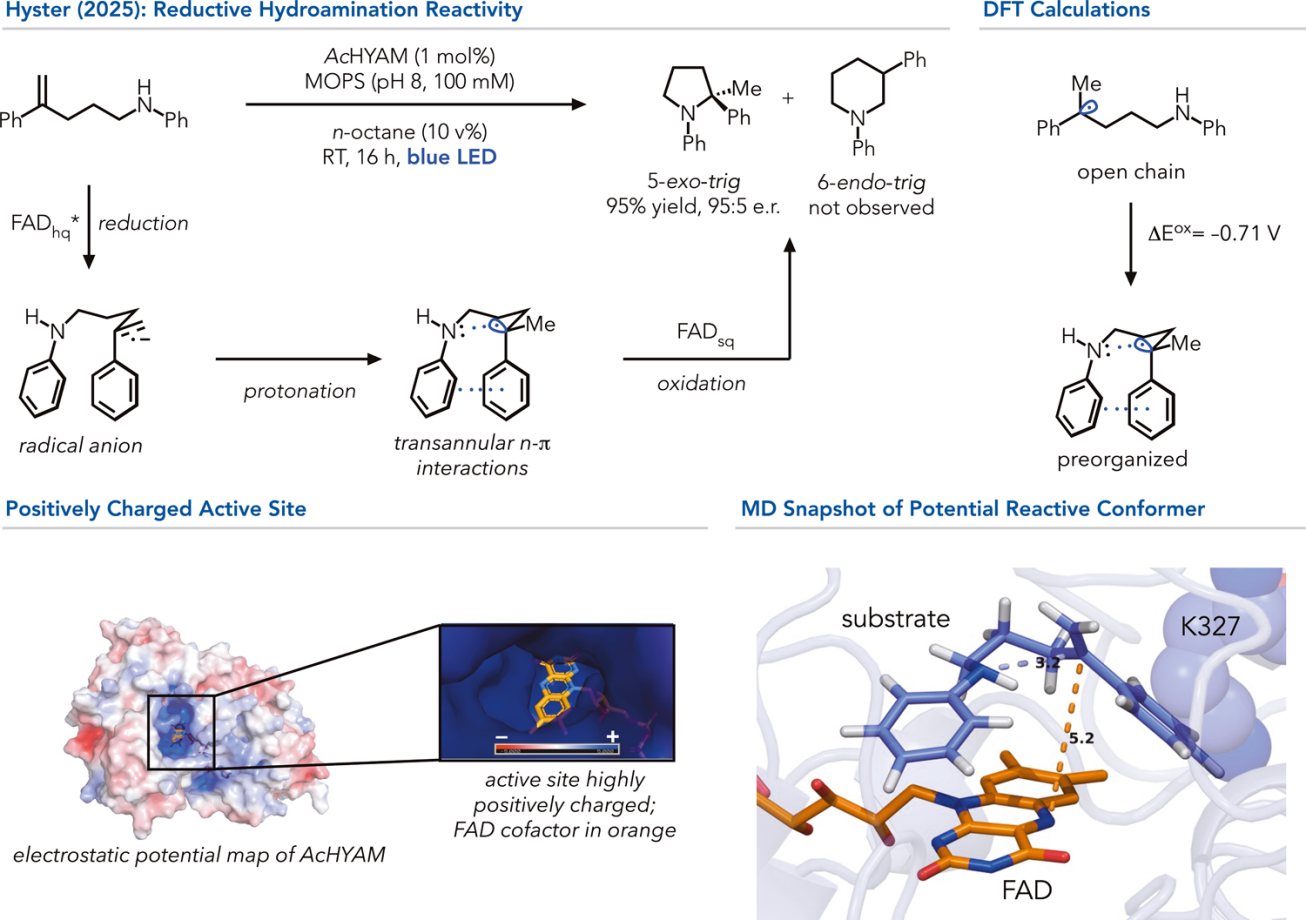
Stereoselective hydroaminations in Baeyer–Villiger monooxygenases (BVMOs) via sequential alkene reduction and through space oxidation potential attenuation for C–N bond formation. The electrostatic potential map explains why the reduction of the styrenyl moiety is possible, and MD simulations reveal a potential reactive conformer in the evolved enzyme.

Carboxylic acids are abundant functional groups often supplied in enantioenriched form, such as canonical amino acids.^80–82^ Tan et al.,^83^ Nishibayashi et al.,^84^ and MacMillan et al. demonstrated that enantioenriched Cbz-protected amino acids could be coupled to Michael acceptors using photoredox catalysts.^85^ However, the stereochemical information from the amino acid is lost in these reactions because they proceed via free radical intermediates. Melchiorre and co-workers demonstrated a novel biocatalytic strategy that transfers the stereochemical information from the carboxylic acid starting material to the product despite proceeding via radical decarboxylative reaction.^86^ They found a deoxyribose-phosphate aldolase (DERA) can catalyze the coupling of cinnamaldehydes with carboxylic acids under UV-light irradiation (Figure 10).^87^ Mechanistically, the catalytic lysine reacts with the aldehyde to form an enzyme-bound iminium, which, upon irradiation, becomes a potent oxidant. This species can oxidatively decarboxylate carboxylic acids to afford an alkyl radical and β-enaminyl radical, which rapidly react to form a C–C bond. They hypothesize that the carboxylic acid binds in a favored orientation prior to oxidation. Because the C–C bond formation is faster than reorientation of the alkyl radical within the active site, the stereochemical information from the starting material is transferred to the product (***type 1***). The retention of enantioselectivity is highly reliant on the protein active site and exemplifies the high degree of control possible within protein frameworks.

**Figure 10:**
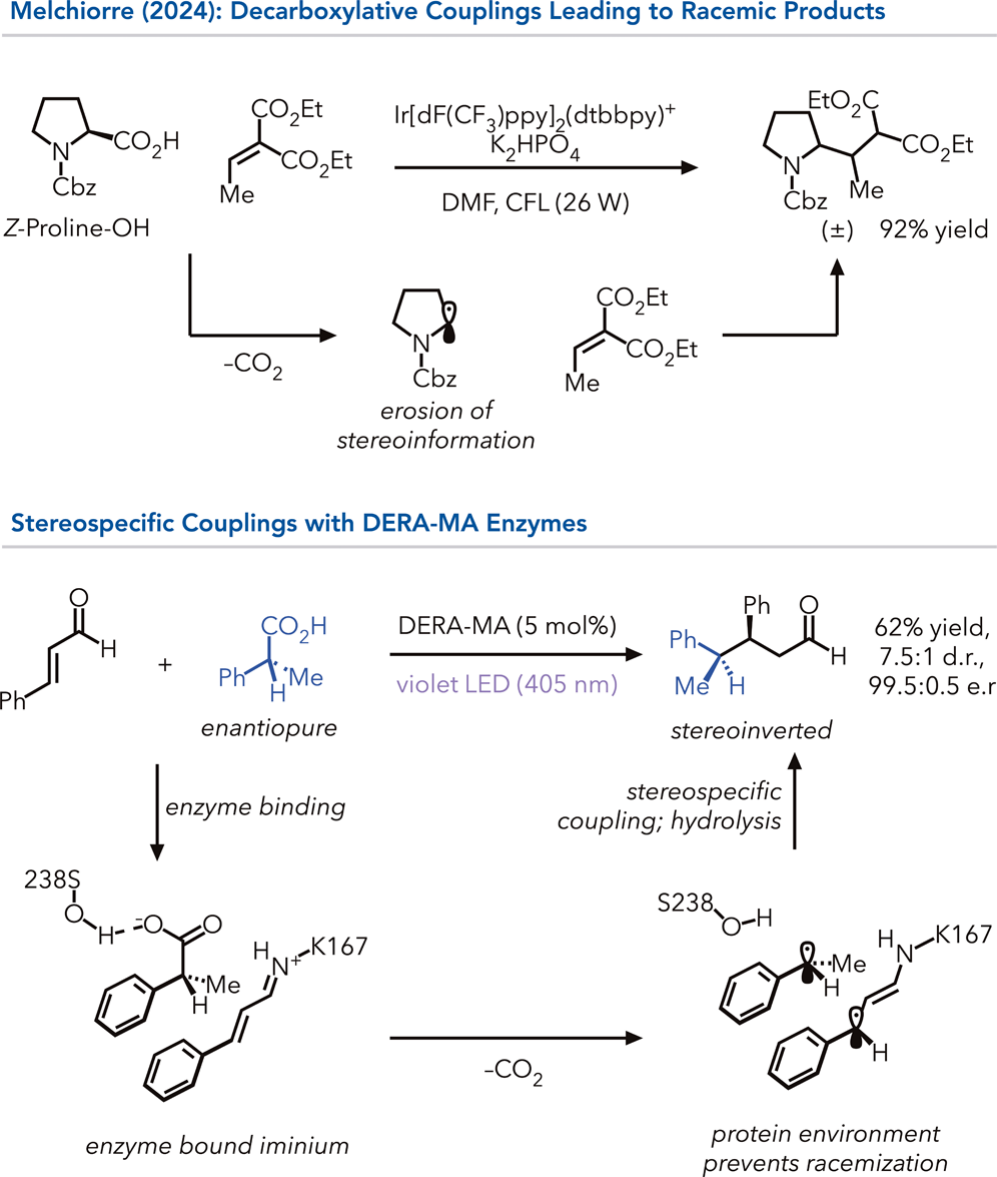
Aldolases for stereospecific oxidative decarboxylation.

## Synergistic Photoenzymes

Enzymes can generate reactive intermediates within their active sites that would be less stable in solution. An example of this phenomenon is pyridoxal-5′-phosphate (PLP) dependent enzymes, which can generate and stabilize electrophilic aminoacrylate and nucleophilic quinonoid species from amino acids. These intermediates can react with various nucleophilic and electrophilic reagents, respectively, to form useful amino acid products (Figure 11).^88^ These enzymes are attractive because they catalytically generate these reactive intermediates, avoiding the stoichiometric activation strategies typically required when using small molecule catalysts.^89^

**Figure 11:**
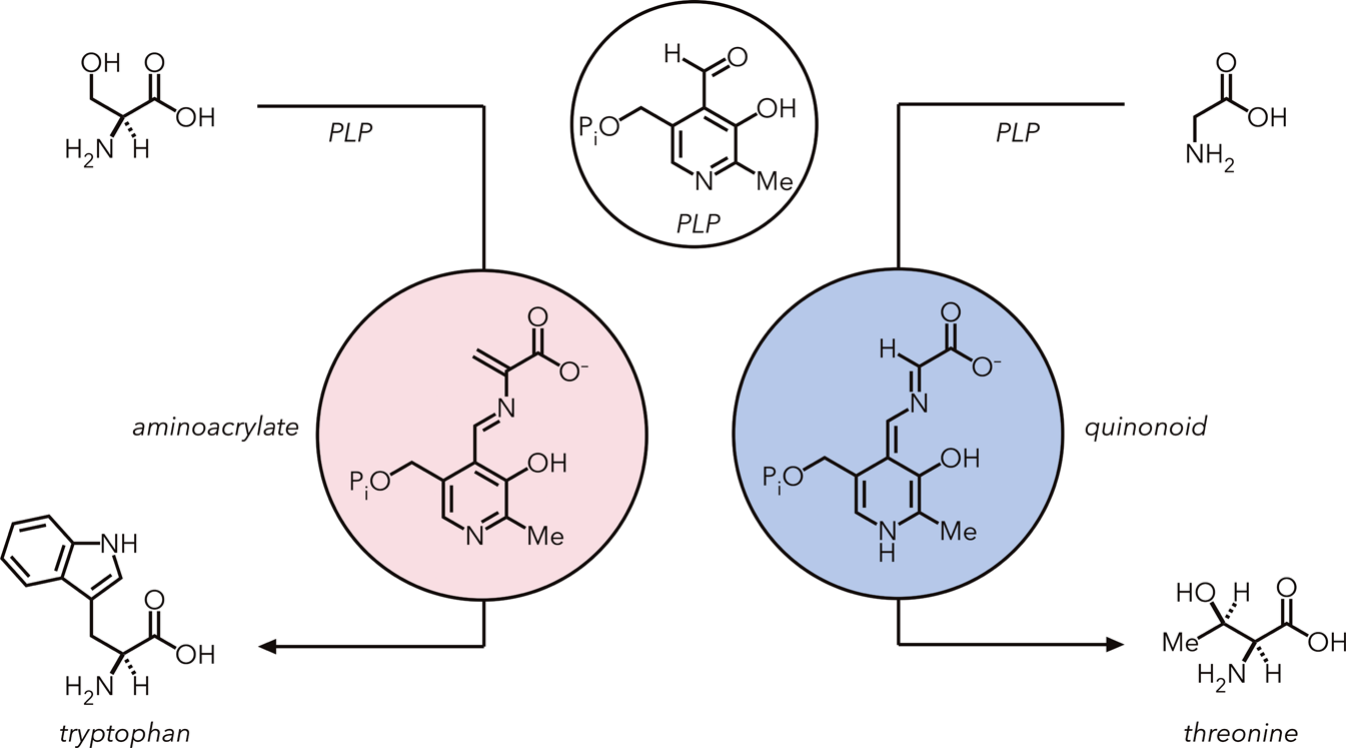
The natural activity of PLP-dependent enzymes enables the formation and stabilization of distinct reactive intermediates within the active site. Left: Intermediate of tryptophan synthase. Right: Threonine synthase intermediate.

Yang, Liu, and co-workers demonstrated that these intermediates can be used to access noncanonical amino acids using non-natural radical intermediates.^90^ In their first study, they used the tryptophan synthase β-subunits from *Pyrococcus furiosus* to generate the electrophilic aminoacrylate species from serine. This species is an electrophilic Michael acceptor and is reported to react with various ionic nucleophiles (Figure 12).^91^ Yang found that when this enzyme is used in combination with Rhodamine B photocatalyst and benzyl trifluoroborate salts, benzylic radicals can be generated, which react with the aminoacrylate for various homophenylalanine products in good yield with excellent enantioselectivity. In addition to serine, the enzyme accepts threonine, enabling the synthesis of noncanonical amino acids bearing three contiguous stereocenters set with high enantio- and diastereoselectivity.^92^

**Figure 12:**
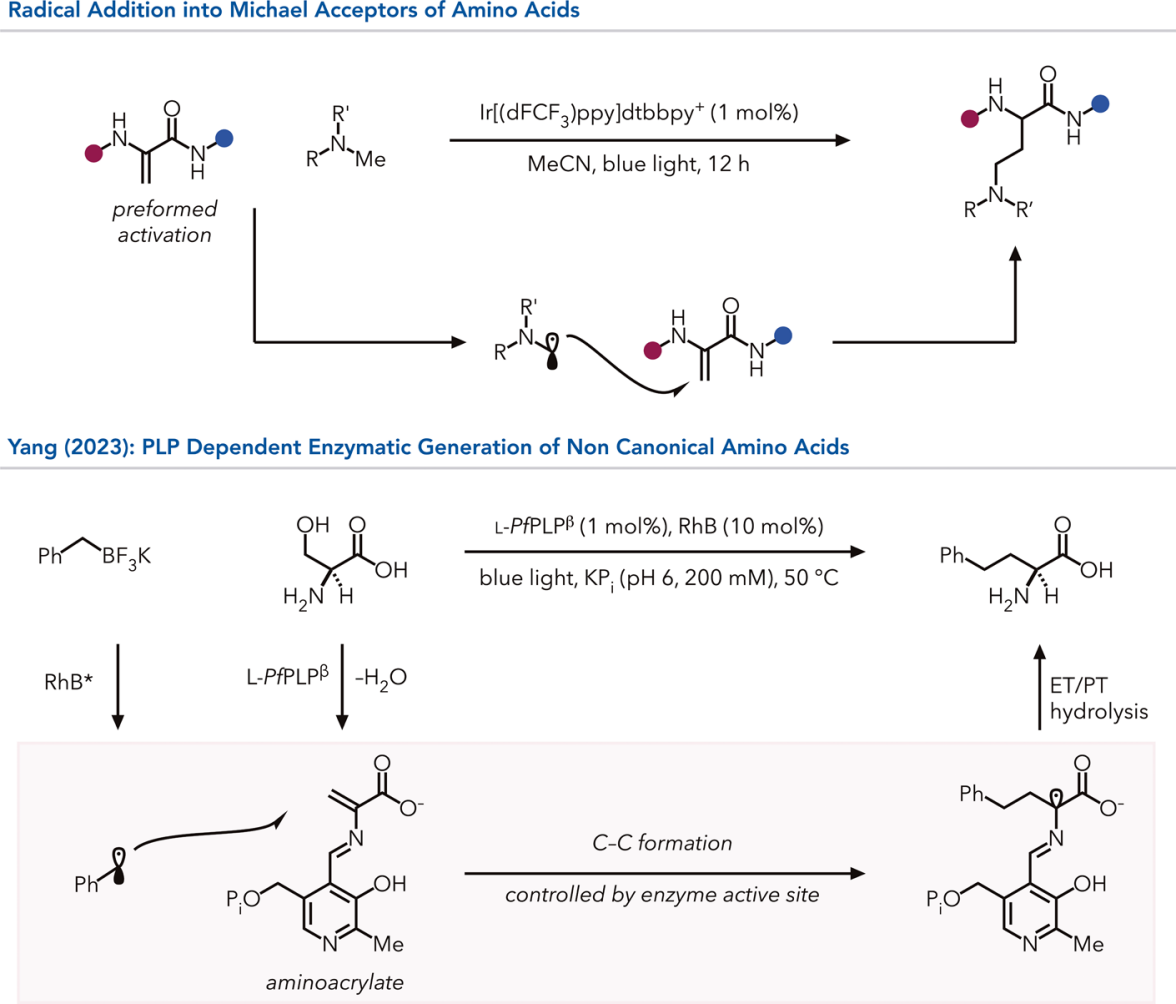
Small-molecule-catalyzed reactivity relies on the preactivation of amino acids for functionalizations. In contrast, PLP-dependent C–C formation forms a covalent, electrophilic intermediate stabilized by the enzyme active site. RhB = rhodamine B.

Related radical coupling can be achieved using the nucleophilic quinonoid species in threonine aldolases. Natively, these proteins catalyze the coupling of aldehydes with small amino acids to afford threonine analogs. Independent studies by Yang and Hyster demonstrated that transient PLP intermediates can capture distinct radical intermediates (Figure 13).^93,94^ Both methods leverage the protein environment of l-threonine aldolase from *Thermotoga maritima* (*Tm*LTA) to generate a quinonoid intermediate. In Yang’s work, the radical is oxidatively generated from benzyl boronic esters, while Hyster exploits reductive radical formation from pyridinium salts. In both cases, the α-amino stereocenter is set with high levels of enantioselectivity.

**Figure 13:**
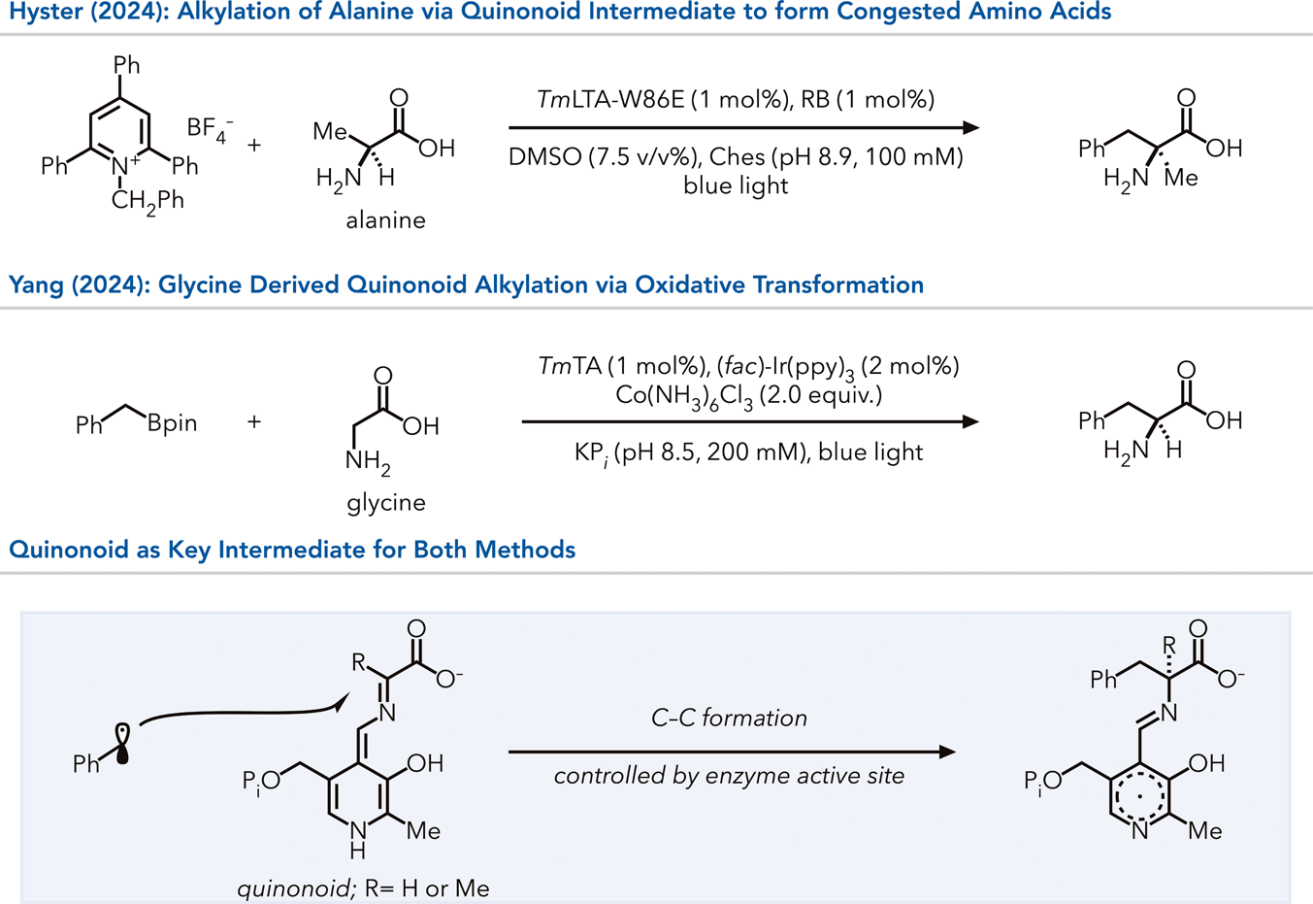
Activation of alanine as described by Hyster for selective couplings that generate noncanonical, congested amino acids. Corollary strategy for glycine functionalization by Yang and Liu for quinonoid based functionalizations. RB = Rose Bengal.

The study of synergistic radical formation and reaction with intermediates that are stabilized by a protein environment are an ongoing effort within the field. While PLP dependent enzymes have highlighted the potential for synergies with photocatalysts, there had been earlier examples with ERED hydroamination reactions that harness similar interactions.^95^ In addition, more recent examples have highlighted the applicability to established Umpolung radical reactivity by Huang that harnesses photocatalysts for its reactivity.^96,97^ Nevertheless, more effort has to be dedicated toward understanding the nature of the precise interactions between the enzymes and photocatalysts, and how electron transfer events are governed by these interactions.

More effort has to be dedicated toward understanding the nature of the precise interactions between the enzymes and photocatalysts, and how electron transfer events are governed by these interactions.

This will ultimately unlock a vast space of radical reactivity with enzymes lacking natural chromophores extending the potential application for challenging synthetic disconnections.

## Conclusion

The highlighted examples showcase the ability of enzymes to facilitate mechanisms and bond formations that are currently unknown to small molecule catalysts. The ability of the protein microenvironment to reveal elusive mechanisms expands the types of transformations that can be used in chemical synthesis. But is there any rational way to explore “new” reactivity? The presented emergent activities are primarily the result of side products observed throughout engineering “known” reactivity. The translation efforts with the goal of higher control inevitably lead to side products, enabling their amplification through directed evolution. Hence, we expect the field to report increasingly unexpected results with increased biocatalytic scaffold implementation for established, non-natural reactivities.

We expect the field to report increasingly unexpected results with increased biocatalytic scaffold implementation for established, non-natural reactivities.

These emergent pathways are crucial to broadening the synthetic capabilities, as modern drug design requires tailored catalysts for their streamlined preparation. However, they can also provide a rebound to small molecule catalyst design and find minimal systems that can help open distinct avenues of catalysis.
